# Analyses of Safety Profile and Homologous Antibody Responses to a Mammalian Cell-Based, MF59-Adjuvanted, A/H5N1, Pandemic Influenza Vaccine across Four Phase II/III Clinical Trials in Healthy Children, Adults, and Older Adults

**DOI:** 10.3390/vaccines9121468

**Published:** 2021-12-11

**Authors:** Eve Versage, Esther van Twuijver, Wim Jansen, Ad Theeuwes, Daphne Sawlwin, Matthew Hohenboken

**Affiliations:** 1 Seqirus USA Inc., Cambridge, MA 02139, USA; matthew.hohenboken@seqirus.com; 2 Seqirus Netherlands B.V., 1105 BJ Amsterdam, The Netherlands; esther.vantwuijver@seqirus.com (E.v.T.); wim.jansen@seqirus.com (W.J.); adrianus.theeuwes@seqirus.com (A.T.); 3 Seqirus Pty Ltd., Melbourne 3052, Australia; daphne.sawlwin@seqirus.com

**Keywords:** influenza, vaccine, pandemic, cell culture, MF59, adjuvant, H5N1

## Abstract

Modern cell culture-based technology eliminates vaccine manufactures reliance on embryonated chicken eggs, which may become compromised during an avian influenza pandemic. Four studies (total N = 6230) assessed the immunogenicity and safety of mammalian cell-based, MF59^®^-adjuvanted, A/H5N1 vaccine (aH5N1c; AUDENZ™) as two doses administered on Days 1 and 22 in children (NCT01776554), adults (NCT01776541; NCT02839330), and older adults (NCT01766921; NCT02839330). Immunogenicity of formulations at 7.5 μg and 3.75 μg antigen per dose were assessed by hemagglutination inhibition and microneutralization assays on Days 1, 22, 43, and 183 or 387. Solicited local and systemic adverse events (AEs) were recorded for 7 days after each vaccination. Unsolicited AEs were collected for 21 days after each vaccination, and serious and other selected AEs were recorded for one year. Antibody responses after two 7.5 μg doses met CBER licensure criteria in all age groups. Overall, an age-related response was evident, with the highest responses observed in children <3 years old. In children, antibody titers met seroconversion criteria 12 months after vaccination. MF59 allowed for antigen dose sparing. Solicited AEs were mild to moderate in nature, of short duration, and less frequent after the second dose than the first, demonstrating a favorable risk-benefit profile.

## 1. Introduction

In recent history, major influenza pandemics occurred in 1918 (A/H1N1), 1957 (A/H2N2), 1968 (A/H3N2), and 2009 (A/H1N1), these events resulted in approximately 45, 1.5, 1.3, and 0.6 million human deaths, respectively [1]. The A/H5N1 influenza virus is highly pathogenic, and has significant pandemic potential. Transmission of avian A/H5N1 from birds to humans is relatively rare; however, mortality rates in the human population are exceptionally high, with 455 of 862 (53%) total laboratory-confirmed A/H5N1 cases reported to the World Health Organization (WHO) between 2003–2020 being fatal [2]. In the event of an outbreak, very few people would possess pandemic strain-specific memory lymphocyte populations and would have no immunity to the virus. Therefore, effective and safe A/H5N1 pandemic vaccines for individuals of all ages are a public health priority.

Pandemic preparedness strategy includes the preparation and licensing of pandemic influenza vaccines in advance of any future pandemic, thereby significantly decreasing the amount of time required to develop and deliver vaccine during the critical early stages of an outbreak. Proactively having vaccines licensed during inter-pandemic periods should facilitate the regulatory approval of strain-matched vaccine during a pandemic. Currently, three A/H5N1 vaccines are licensed by the U.S. Food and Drug Administration for possible use during a future pandemic, all of which are manufactured using traditional egg-based technology [3]. The assessment of a (now U.S. licensed) mammalian cell-based, A/H5N1, pandemic vaccine described in this report was conducted as part of a program designed by Seqirus USA Inc. in partnership with the U.S. Biomedical Advanced Research and Development Authority (BARDA) to develop a cell-based vaccine to support national and international pandemic preparedness needs.

Modern cell culture-based technology eliminates vaccine manufactures reliance on embryonated chicken eggs, the quality and quantity of which may well be compromised during an avian influenza pandemic, resulting in critical delays to antigen production and vaccine distribution. The propagation of virus in cell culture avoids the occurrence of egg-adaptive hemagglutinin (HA) mutations, which may decrease vaccine effectiveness [4,5,6,7,8]. Cell-based vaccines are suitable for individuals with egg allergy. Cell-based systems also allow for reduced risk of contamination, and antigen production from strains of influenza that do not grow well in eggs [9,10,11,12,13,14].

Decreasing the amount of antigen per dose required to meet the pandemic influenza vaccine licensure criteria (i.e., dose sparing)—as established by the Center for Biologics Evaluation Research and Review (CBER; USA) [15], and the Committee for Medicinal Products for Human Use (CHMP; Europe) [16]—is a valuable attribute of a pandemic vaccine. Being able to induce antibody titers considered to be protective by the regulatory authorities using reduced quantities of HA per dose, means that more vaccine doses can be manufactured and ultimately more individuals protected against the pandemic; thus, dose sparing maximizes the capacity and speed of antigen production. In response to the 2009 A/H1N1 pandemic, the WHO recommended that A/H1N1 vaccines should be adjuvanted, in order to allow for dose sparing and therefore, maximal population coverage [17]—one such adjuvant is the oil-in-water emulsion, MF59^®^ (Seqirus UK Ltd.). As well as increasing antigen-specific antibody responses to many strains of influenza with proven dose sparing potential in recipients of all ages, MF59 has been shown to enhance long-term antibody persistence, and importantly, to promote the production of cross-reactive antibodies able to provide a degree of cross-clade heterologous immunity [18,19,20,21,22,23,24,25,26,27,28,29]. Many pandemic influenza strains require the use of an adjuvant to enhance the host immune response and induce antibody titers considered to be protective. Several clinical trials have shown MF59 to decrease the quantity of antigen required per dose for A/H1N1 [23,24,28,30,31,32,33], A/H3N2 [34], and A/H7N9 [18] pandemic vaccines. When extrapolated and considered in relation to national pandemic immunization programs, such dose sparing potential is highly desirable in helping to achieve maximal population coverage.

Here we report immunogenicity and safety analyses of a mammalian cell-based, MF59-adjuvanted, A/H5N1 vaccine across four clinical trials of similar design, comparing responses to 7.5 μg (full-dose) and 3.75 μg (half-dose) formulations in recipients of all ages.

## 2. Materials and Methods

### 2.1. Vaccines

The investigational, mammalian cell-based, MF59-adjuvanted, monovalent, inactivated vaccine (aH5N1c) included purified A/H5N1 (turkey/Turkey/1/2005-like strain (NIBRG-23)) HA and neuraminidase cell surface antigens, and was prepared by cell cultivation in Madin-Darby canine kidney (MDCK) cells [35]. One 0.5-mL dose of aH5N1c contained 7.5 μg HA antigen and 0.25 mL MF59 (full-dose formulation; as approved for AUDENZ™ (Seqirus USA Inc., Cambridge, MA, USA)). One 0.25-mL dose contained 3.75 μg HA and 0.125-mL MF59 (half-dose formulation). MF59 contained 9.75 mg squalene, 1.18 mg polysorbate 80, and 1.18 mg sorbitan trioleate per 0.25 mL. Vaccines were administered as single intramuscular injections in the nondominant arm, or anterolateral thigh in young children.

### 2.2. Study Design and Objectives

The integrated summary presented in this report is derived from four separate clinical trials conducted between 2013 and 2017: (1) Study A (NCT01776554; V89_11), Phase II, healthy children 6 months–17 years of age (N = 662); (2) Study B (NCT01776541; V89_04), Phase II, healthy adults 18–64 years (N = 979); (3) Study C (NCT02839330; V89_18), Phase III, healthy adults ≥ 18 years (N = 3196); (4) Study D (NCT01766921; V89_13), Phase II, healthy older adults ≥ 65 years of age (N = 1393). Full details of the design and methodology of the individual trials have been published previously [36,37], and are also available at: https://clinicaltrials.gov/ (accessed 27 May 2021). Study protocols were approved by the Ethics Review Committees of participating centers. All studies were conducted in accordance with Good Clinical Practice guidelines and the Declaration of Helsinki [38,39]. Written informed consent was obtained from subjects or their parents/guardians prior to enrollment. Subjects in Studies A, B, and D received either two full-doses or two half-doses of aH5N1c administered three weeks apart on Day 1 and Day 22. Subjects in Study C received either two full-doses of aH5N1c or placebo administered on Day 1 and Day 22. The primary immunogenicity endpoints for each study were antibody responses on Day 43, as assessed by hemagglutination inhibition (HI) assay and expressed as the percentages of subjects achieving HI titers ≥ 1:40.

### 2.3. Study Participants

Study A enrolled 662 children 6 months–17 years of age. Study B enrolled 979 adults 18–64 years of age. Study C enrolled 3196 adults ≥ 18 years of age. Study D enrolled 1393 adults ≥ 65 years of age. Across the four studies, all participants were generally in good health. Full details of the inclusion and exclusion criteria for each trial are published [36,37]. The main exclusion criteria across the four studies were: presence of serious chronic or progressive disease; any acute illness within 3 days of receiving study vaccine; a history of impaired immune function; any progressive or severe neurological disorder; allergy to latex or any vaccine component; cognitive impairment or psychiatric disease; pregnancy or breastfeeding; previously confirmed or suspected A/H5N1 influenza disease; prior receipt of any A/H5N1 vaccine; receipt of any other influenza vaccine within 7 (Study C) or 60 (Studies A, B, and D) days prior to enrollment; receipt of any licensed inactivated vaccines within 14 days prior to enrolment; receipt of any licensed live vaccines within 28 days prior to enrolment; receipt of any other investigational product within 30 days prior to study Day 1; a history of drug or alcohol abuse; planned surgery during the study period; body temperature ≥ 38.0 °C; and body mass index ≥ 35 kg/m^2^.

### 2.4. Immunogenicity Assessment

Blood samples were obtained by venipuncture using vacuum collection tubes and centrifuged immediately at 1500× *g* for 10 min, sera were stored at ≤−18 °C. Homologous antibody titers against the vaccine antigen strain (A/turkey/Turkey/1/2005) were measured by HI and microneutralization (MN) assays (HI assay alone in Study C) according to standard procedure on Days 1, 22, 43, and 183 or 387. Immunogenicity was expressed as geometric mean antibody titers (GMTs), the geometric mean ratios (GMRs) of GMTs, the percentages of subjects with titers ≥ 1:40, and the percentages of subjects achieving seroconversion. Seroconversion in the individual vaccine recipient was defined as a negative pre-vaccination HI titer of <1:10 to a positive post-vaccination titer of ≥1:40, or a minimum four-fold increase where pre-vaccination titers were ≥1:10. HI titers below the detection limit of 10 were arbitrarily assigned to half that limit (5) for the purpose of analysis.

### 2.5. Safety Assessment

The safety data from the three adult trials (Studies B, C, and D) were combined to create an integrated analysis of data, as these three trials were similar in overall design, inclusion/exclusion criteria, and safety data collection methods. In all four trials, including the pediatric trial, subjects were observed for a minimum of 30 min after each vaccination to monitor for immediate adverse reactions. Solicited local and systemic adverse events (AEs) were recorded with diary cards for 7 consecutive days after each vaccination, either by the subjects themselves or the subjects’ parents/legal guardians. Solicited local AEs included ecchymosis, erythema, induration, and tenderness/pain at the site of injection. Solicited systemic AEs included headache, arthralgia, chills, fatigue, malaise, myalgia, nausea, sweating, loss of appetite/altered eating habits, irritability, and fever ≥ 38 °C. Unsolicited AEs were recorded for 21 days after each vaccination (Day 1–43). Serious adverse events (SAEs), adverse events of special interest (AESI), new onset of chronic disease (NOCD), AEs leading to study or vaccine withdrawal, and AEs requiring medical attention were recorded throughout the entire duration of the studies.

### 2.6. Statistical Analyses

Immunogenicity endpoints were analyzed based on current CBER pandemic influenza vaccine licensure criteria, and former CHMP criteria (which were in place at the time the trials were conducted) [15,16]. The following CBER licensure criteria applied: the lower bound of the 2-sided 95% or 97.5% confidence interval (CI) for the proportion of subjects achieving HI seroconversion should be ≥40% for children (≤17 years of age) and adults (18–64 years of age) and ≥30% for older adults (≥65 years of age; herein termed the ‘seroconversion criterion’); the lower bound of the 2-sided 95% or 97.5% CI for the proportion of subjects achieving HI titers ≥ 1:40 should be ≥70% for children and adults, and ≥60% for older adults (termed the ‘≥1:40 criterion’). The following CHMP licensure criteria applied: the proportion of subjects achieving HI seroconversion should be >40% for adults (18–59 years of age) and >30% for older adults (≥60 years of age; ‘seroconversion criterion’); GMRs should be >2.5 for adults and >2.0 for older adults (‘GMR criterion’); and the proportion of subjects achieving HI titers ≥ 1:40 should be >70% for adults and >60% for older adults (‘≥1:40 criterion’). Because CHMP criteria were not defined for children, adult criteria were applied to the pediatric population. In the studies where two vaccine formulations were assessed, 97.5% CIs were applied to the CBER criteria to adjust for multiplicity. Safety data were evaluated descriptively and expressed as the percentages or numbers of subjects with AEs in a group. Subjects were excluded from Per Protocol analysis for immunogenicity if major protocol deviations occurred. Statistical analyses were performed using SAS version 9.3^®^ software (SAS Institute Inc., Cary, NC, USA).

## 3. Results

Of the 5568 adult subjects enrolled into Studies B, C and D, 3579 received the full-dose formulation; 47% (1683/3579) and 53% (1896/3579) were 18–64 and ≥65 years old, respectively. All 3579 adult subjects received a first dose, 97% (3470/3579) received a second dose. The three adult trials were completed by 93% (3344/3579) of subjects. In Study C, placebo was administered to 398 adults and 398 older adults. Of the 662 children enrolled into Study A, 329 were exposed to the full-dose formulation, 97% (318/329) of whom received a second dose. Overall, 94% of subjects (315 of 329, full-dose group; 307 of 329, half-dose group) completed the study on Day 387. The baseline characteristics of the study populations are shown in Table 1.

Within individual Studies A, B and D, subjects in the full-dose and half-dose groups were similar in terms of age, gender, race, ethnicity, body mass index, and the receipt of seasonal influenza vaccine within the previous 12 months. The demographics of the adult and older adult populations in Study C are presented in Table 1, with a notably higher proportion of older adults having previously received seasonal influenza vaccine. Both Full Analysis Set (FAS) and Per Protocol Set (PPS) data were used to evaluate immunogenicity.

### 3.1. Immunogenicity

**Figure 1:**
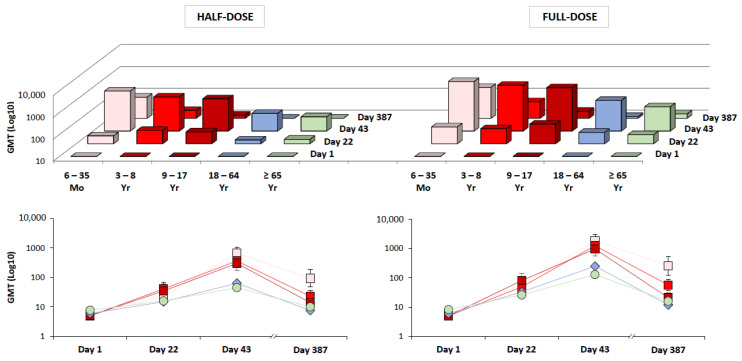
Geometric mean antibody titers (GMTs; hemagglutination inhibition assay) at baseline (Day 1), three weeks after first dose (Day 22), and three weeks (Day 43) and twelve months (Day 387) after second dose administration. Data for subjects 6 months–17 years, 18–64 years, and ≥65 years of age derived from Studies A, B and D, respectively (Full Analysis Set data). Three-dimensional column graphs and line graphs show identical GMT data in response to half-dose and full-dose formulations. Line graphs show 95% CIs for Study A and Study B data (subjects 6 months–64 years of age), and 97.5% CIs for Study D data (subjects ≥ 65 years of age).

**Figure 2:**
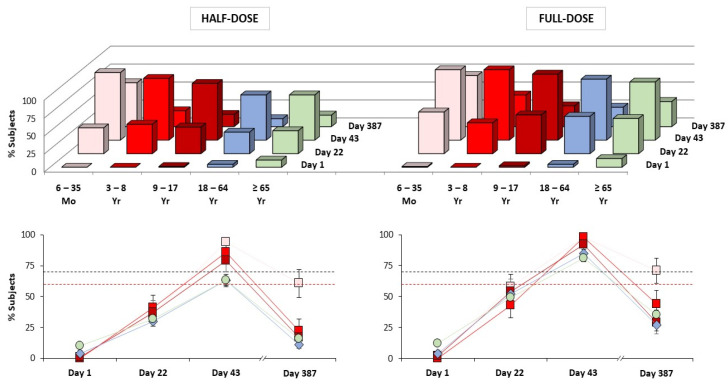
Percentages of subjects (95% CI) achieving hemagglutination inhibition antibody titers ≥ 1:40 at baseline (Day 1), three weeks after first dose (Day 22), and three weeks (Day 43) and twelve months (Day 387) after second dose administration. Data for subjects 6 months–17 years, 18–64 years, and ≥65 years of age derived from Studies A, B and D, respectively (Full Analysis Set data). Three-dimensional column graphs and line graphs show identical data in response to half-dose and full-dose formulations. Black broken lines indicate CBER and CHMP thresholds (70%) for HI ≥ 1:40 licensure criteria in adults, red broken lines indicate CBER and CHMP thresholds (60%) for older adults (refer to Table 4 for additional information regarding CBER and CHMP licensure criteria).

Immunogenicity analyses against the A/H5N1 vaccine antigen strain, turkey/Turkey/1/2005 by HI assay are shown in Figure 1 and Figure 2, and Table 2, Table 3 and Table 4.

In all age groups, two doses of the MF59-adjuvanted vaccine formulation containing 7.5 μg antigen (full-dose) met all CBER and all CHMP licensure criteria three weeks after second dose administration (Day 43; Figure 2; Table 4). In pediatric subjects, seroconversion criteria were met three weeks after first dose (Day 22; Table 4). For all age groups, GMTs were higher in response to the full-dose than the half-dose formulation on Day 43 and Day 387 (Figure 1). The CHMP criterion for GMR was met by the full-dose formulation after a single dose (Day 22) in all age groups (Table 3). Overall, a second dose was beneficial, resulting in further increases in antibody titers among vaccine recipients of all ages. An age-related response was evident in both full-dose and half-dose recipients; the highest antibody responses were observed in children 6 months to <3 years of age, with 98% (95% CI: 92–100%) of this population achieving HI titers ≥ 1:40 after two doses (Day 43; GMT 1842, data not shown). Although antibody responses in subjects ≥ 65 years of age met both CBER and CHMP licensure criteria, consistent with the principle of immunosenescence—responses were lower in older adults (HI ≥ 1:40, 81–84%; GMT, 98–129) than in adults (HI ≥ 1:40, 85–94%; GMT, 171–265).

Long-term antibody persistence was observed up to one year (Day 387) after immunization with both full-dose and half-dose formulations (Figure 1 and Figure 2; note, Day 183 persistence data not shown because antibody titers were not assessed on Day 183 in all four studies). Although lower than the titers observed on Day 43, HI titers on Day 387 persisted above baseline levels in recipients of all ages. Long-term persistence was highest in the pediatric population, and in response to the full-dose formulation. Pediatric HI titers on Day 387 were sufficiently high to continue meeting the CBER criterion for seroconversion, and the CHMP criteria for seroconversion and GMR (Figure 2). Although long-term persistence was observed in adults and older adults, these antibody titers did not meet either CBER or CHMP criteria. The receipt of seasonal influenza vaccine prior to enrollment had no influence on whether licensure criteria were met or not. Cross-reactive antibody responses were observed against five heterologous A/H5N1 strains, these data will be published in a separate report. Overall, HI results were supported by similar MN data; generally, MN antibody titers were higher than HI titers.

### 3.2. Safety

Overall, solicited local and systemic AEs were mild to moderate in nature and resolved spontaneously within a few days of onset. Solicited local and systemic AEs occurred less frequently after the second dose than the first dose in all age groups and across all vaccine groups (safety data for half-dose recipients (not shown in Table 5, Table 6, Table 7 and Table 8) have been published previously [36,37]). Solicited local and systemic AEs were experienced less frequently by older adults than adults. The most frequent solicited local AE in adults and older adults after receipt of the full-dose formulation and placebo was injections site pain, experienced by 39–65% of the full-dose group and by 10–20% of the placebo group; severe pain was rare at 0–<1% (Table 5).

Injections site pain was also the most frequent local AE among the pediatric population (only full-dose data presented; Table 5), occurring at 56% and 68% in children 6 months–5 years and 6–17 years of age, respectively; severe pain was rare at 1–2%. After pain, the next most frequent local AEs across all age groups were erythema (at 3% in children < 6 years old) and induration (at 2% in both children 6–17 years old and older adults receiving the full-dose formulation). The most frequent solicited systemic AEs in adults receiving the full-dose were fatigue and headache, both occurring at 25%, and at similar rates in the placebo control groups (Table 6).

Fatigue was most common in older adults at 19% in both full-dose and placebo groups. Loss of appetite was the most frequently reported AE in children < 6 years of age, experienced by 18% of subjects. Myalgia was the most frequently reported AE in children 6–17 years of age, experienced by 30% of subjects. Fever ≥ 38.0 °C was experienced by 16% and 4% of children < 6 and 6–17 years of age, respectively; considerably lower rates of fever were observed in adults (1% full-dose; 2% placebo) and older adults (1% full-dose; <1% placebo; Table 6). Severe fever ≥ 40 °C occurred in 1% of children < 6 years old, and in <1% of adults receiving the full-dose formulation.

The nature, frequency, and severity of unsolicited AEs (including SAEs, AESI, NOCD, AEs requiring medical attention, and AEs leading to withdrawal) did not raise any safety signal in any age group or any other subgroups analyzed. From Day 1–43, unsolicited AEs occurred at similar rates in children (26%), adults (23%), and older adults (28%) receiving the full-dose formulation (Table 7); for adults and older adults, overall rates of unsolicited AEs were similar in the vaccine (23% and 28%, respectively) and placebo (22% and 24%, respectively) groups.

Incidence of AESI occurring throughout the entire duration of the studies were similar in 18–64-year-old vaccine (<1%) and placebo (0%) recipients, and in ≥65 year-old vaccine (<1%) and placebo (2%) recipients (Table 7). Likewise, incidence of NOCD were similar in 18–64-year-old vaccine (6%) and placebo (5%) recipients, and in ≥65 year-old vaccine (13%) and placebo (13%) recipients. During the entire duration of the studies, unsolicited SAEs occurred more frequently in older adults (9%) than in adults (3%) or in children (2%). Vaccine-related AEs were infrequent and occurred at similar rates in the full-dose (4–8% across age groups) and placebo (6–7%) groups (Table 8).

The most common vaccine-related AE was injection site bruising, occurring at 2% in the adult placebo group, and at 2% in the older adult full-dose and placebo groups. Vaccine-related SAEs were very rare at 0–<1% across age groups in both vaccine (spontaneous abortion, n = 1) and placebo (polymyalgia rheumatica, n = 1; immune thrombocytopenic purpura, n = 1) recipients (Table 7). No cases of anaphylactic reaction occurred during any of the trials. AEs leading to death occurred at <1% in the adult full-dose group, the older adult full-dose group, and the older adult placebo group. No pediatric deaths occurred during the course of Study A. No adult deaths were vaccine-related, and the frequency of these events was in line with U.S. mortality statistics for the age groups in question [40]. No safety signals were detected in any age group following administration of either full-dose or low-dose aH5N1c formulations.

## 4. Discussion

The present study analyzed the immunogenicity and safety of a mammalian cell-based, MF59-adjuvanted, A/H5N1, pandemic vaccine across four individual clinical trials of similar design. Full-dose (7.5 μg) and half-dose (3.75 μg) formulations were assessed in recipients of all ages as part of a program designed to advance national and international pandemic strategy and preparedness [41]. Overall, aH5N1c was found to be highly immunogenic; while a single full-dose induced clinically significant antibody titers, a two full-dose priming series was identified as optimal for the induction of early (up to three weeks post-second dose) and long-term (up to one year post-first dose) antibody responses in recipients of all ages (Figure 1 and Figure 2).

The present study found both full-dose and half-dose aH5N1c formulations to be highly immunogenic, with two full-doses meeting all licensure criteria in all age groups; an age-related response was evident, with the highest antibody titers observed in children < 3 years of age. The immunogenicity and safety profiles of the same vaccine construct containing different virus subtypes (A/H1N1, A/H3N2, and A/H7N9) have been characterized in a number of trials [18,23,34,42,43]. The findings from the four aH5N1c trials described in the present study are generally consistent with the results of previous studies assessing the immunogenicity of mammalian cell-based, MF59-adjuvanted pandemic vaccines containing 7.5 μg and 3.75 μg HA antigen per dose [18,23,34,42,43].

Assessment of cell-based A/H3N2 vaccine in children, adults, and older adults [34], found a similar age-related response to that observed in the present study, with the highest antibody titers occurring in adolescents, and the lowest responses occurring in older adults. A single MF59-adjuvanted, 3.75 μg A/H3N2 dose met all CBER and CHMP licensure criteria in recipients of all ages. The immunogenicity of a cell-based A/H7N9 vaccine created using synthetic seed technology was assessed in adult subjects alone [18]; formulations containing 7.5 μg and 3.75 μg antigen per dose induced significant and potentially protective antibody titers after two doses. Although not directly comparable to aH5N1c due to being egg—rather than cell-based, trials of MF59—adjuvanted A/H5N1 pandemic vaccine do highlight the benefits of adjuvantation for all age groups [19,21,29,44,45]. Czajka et al. assessed the immunogenicity of 7.5 μg and 3.75 μg A/H5N1 doses in adults and older adults [21], finding the low-dose to be non-inferior to the high-dose formulation in terms of vaccine antigen strain-specific antibody responses; three and two CHMP criteria were met by adults and older adults in response to two 3.75 μg doses, respectively.

All four aH5N1c studies included in this review observed antibody persistence up to one year after full-dose and half-dose aH5N1c administration in subjects of all ages, with pediatric titers persisting at levels high enough to continue to meet CBER and CHMP licensure criteria. These findings are supported by similar long-term persistence data in children receiving egg-based A/H5N1 vaccine at 7.5 μg per dose [45], and adults and older adults receiving cell-based A/H1N1 vaccine at 7.5 μg and 3.75 μg per dose [23]. In line with the results of the present study, long-term antibody persistence in response to the A/H5N1 vaccine was highest in the youngest group of children, 6–36 months of age [45]. Likewise, long-term persistence was higher in adults than in older adults one year after receipt of A/H1N1 vaccine [23]. The assessment of a one-year booster dose was not included in the design of the four aH5N1c studies analyzed for this report. However, the immunogenicity of MF59-adjuvanted, one-year booster doses containing antigen homologous to the priming strain have been assessed in previous studies [23,45], the results of which demonstrate booster administration to be highly immunogenic in recipients of all ages, inducing high titers of both vaccine antigen-specific and cross-reactive antibodies. Overall, the safety data presented in this report are in agreement with those of previous comparable trials of MF59-adjuvanted A/H5N1 and A/H1N1 pandemic vaccines in children, adults, and older adults [19,21,23,29,42,43,44,45].

Possible limitations to the four aH5N1c clinical trials analyzed for this report include the absence of A/H5N1 non-adjuvanted vaccine control groups; however, this was previously evaluated in a Phase I study documenting the immunological benefits of MF59 [46]. In conclusion, aH5N1c was highly immunogenic and induced long-term antibody persistence in recipients of all ages, these data demonstrate favorable safety and risk-benefit profiles for this vaccine. The successful development of effective and safe vaccines manufactured using cell-based, high-volume, high-yield technology represents a major advance towards meeting the objective of enabling governments and health authorities to meet the global demand for vaccine in the event of an A/H5N1 influenza pandemic.

## Figures and Tables

**Table 1:** Population demographics and baseline characteristics in aH5N1c vaccine recipients.

		6 Mo–17 y Half-Dose Study A (n = 288)	6 Mo–17 y Full-Dose Study A (n = 289)	18–64 y Half-Dose Study B (n = 440)	18–64 y Full-Dose Study B (n = 451)	18–64 y Full-Dose Study C (n = 1116)	≥65 y Full-Dose Study C (n = 1133)	≥65 y Half-Dose Study D (n = 664)	≥65 y Full-Dose Study D (n = 673)
Age in Years (Mean ± SD)		6.8 ± 4.5	6.6 ± 4.7	39 ± 14	39 ± 13	44 ± 13	72 ± 5.6	71 ± 4.8	71 ± 5.1
Male:Female (%)		51:49	55:45	46:54	42:58	42:58	46:54	41:59	42:58
Race:	American Indian/Native Alaskan (%)	0	<1	<1	<1	<1	<1	0	<1
	Asian (%)	74	72	21	20	2	<1	33	34
	Black/African American (%)	3	3	18	20	20	7	2	<1
	Native Hawaiian/Pacific Islander (%)	0	0	<1	0	<1	<1	0	0
	White (%)	21	23	59	59	77	92	65	64
	Other (%)	2	1	<1	<1	<1	<1	<1	<1
Ethnic Origin:	Hispanic/Latino (%)	4	4	20	21	11	3	2	2
	Non-Hispanic/Non-Latino (%)	96	96	80	79	88	96	98	98
	Not Reported/Unknown (%)	0	0	0	0	1	1	0	0
Body Mass Index (Mean ± SD)		18 ± 3.7	18 ± 3.5	26 ± 4.3	26 ± 4.3	27 ± 4.3	28 ± 4.0	27 ± 4.2	26 ± 4.0
Country:	USA (%)	26	28	58	59	100	100	43	41
	Thailand (%)	74	72	19	19	0	0	33	34
	Australia (%)	0	0	23	22	0	0	13	13
	New Zealand (%)	0	0	0	0	0	0	12	12
Previous Influenza Vaccination * (%)		16	15	20	21	36	71	55	56

Mo, months of age. y, years of age. SD, standard deviation. Body Mass Index: kg/m^2^. Studies A, B, and D, Full Analysis Set data. Study C, Per Protocol Set data. * Receipt of seasonal influenza vaccine within past 12 months. Placebo group data (not shown) similar to that of corresponding full- or half-dose vaccine groups. Sum of country percentage values > 100% for ≥65-year half-dose group, due to rounding of data to nearest whole numbers.

**Table 2:** Percentages (95% ^†^ or 97.5% ^‡^ CI) of subjects achieving seroconversion in response to full-dose formulation, as assessed by hemagglutination inhibition assay three weeks after first dose (Day 22), and three weeks after second dose (Day 43) administration.

Age Range	Day 22	Day 43
6–35 Months (Study A)	**58%** (46–69 ^†^) n = 83	**99%** (93–100 ^†^) n = 83
3–8 Years (Study A)	**43% **(33–54 ^†^) n = 95	**98%** (92–100 ^†^) n = 93
9–17 Years (Study A)	**53% **(43–63 ^†^) n = 100	**92%** (85–96 ^†^) n = 100
18–64 Years (Study B)	**48%** (43–54 ^‡^) n = 464	**83%** (78–87 ^‡^) n = 451
18–64 Years (Study C)	**40% **(38–43 ^†^) n = 1115	**80%** (77–82 ^†^) n = 1076
≥65 Years (Study C)	24% (22–27 ^†^) n = 1130	**54%** (51–57 ^†^) n = 1080
≥65 Years (Study D)	**36%** (32–40 ^‡^) n = 681	**74%** (70–77 ^‡^) n = 673

Seroconversion in baseline seronegative subjects defined as pre-vaccination HI titer < 1:10 to post-vaccination HI titer ≥ 1:40. Seroconversion in baseline seropositive subjects defined as pre-vaccination HI titer ≥ 1:10 to ≥4-fold increase in post-vaccination HI titer. Bold red text, either CBER and/or CHMP ≥ 1:40 licensure criteria met (also refer to Table 4). Studies A, B, and D, Full Analysis Set data. Study C, Per Protocol Set data.

**Table 3:** Geometric mean ratios (GMRs; 95% ^†^ or 97.5% ^‡^ CI) in response to full-dose formulation, as assessed by hemagglutination inhibition assay three weeks after first dose (Day 22), and three weeks after second dose (Day 43) administration.

Age Range	Day 22:Day 1	Day 43:Day 1
6–35 Months (Study A)	**12 **(7.3–19 ^†^) n = 84	**302** (192–476 ^†^) n = 84
3–8 Years (Study A)	**9.8 **(6.0–16 ^†^) n = 95	**249** (153–404 ^†^) n = 93
9–17 Years (Study A)	**15** (8.8–27 ^†^) n = 102	**186** (105–328 ^†^) n = 102
18–64 Years (Study B)	**5.4 **(4.6–6.3 ^†^) n = 464	**41** (34–49 ^†^) n = 451
18–64 Years (Study C)	**3.8 **(3.6–4.1 ^†^) n = 1115	**13 **(12–14 ^†^) n = 1076
≥65 Years (Study C)	**2.1 **(2.0–2.3 ^†^) n = 1130	**4.9 **(4.6–5.2 ^†^) n = 1080
≥65 Years (Study D)	**3.2 **(2.8–3.7 ^‡^) n = 681	**16 **(13–19 ^‡^) n = 673

GMR, geometric mean ratio, defined as the ratio of group geometric mean antibody titers (GMTs) on two study days (e.g., Day 22 GMT:Day 1 GMT). Bold red text, CHMP GMR licensure criterion met (also refer to Table 4). Studies A, B, and D, Full Analysis Set data. Study C, Per Protocol Set data.

**Table 4:** Overview of influenza vaccines licensure criteria met in response to full-dose formulation, as assessed by hemagglutination inhibition assay three weeks after first dose (Day 22), and three weeks after second dose (Day 43) administration.

Age Range	Clinical Trial	CBER (USA)				CHMP (Europe)					
		Day 22		Day 43		Day 22			Day 43		
		≥1:40	SC	≥1:40	SC	≥1:40	SC	GMR	≥1:40	SC	GMR
6–35 Mo	Study A	**–**	**✓**	**✓**	**✓**	**–**	**✓**	**✓**	**✓**	**✓**	**✓**
3–8 Yr	Study A	**–**	**–**	**✓**	**✓**	**–**	**✓**	**✓**	**✓**	**✓**	**✓**
9–17 Yr	Study A	**–**	**✓**	**✓**	**✓**	**–**	**✓**	**✓**	**✓**	**✓**	**✓**
18–59 Yr	Study C	**–**	**–**	**✓**	**✓**	**–**	**✓**	**✓**	**✓**	**✓**	**✓**
18–60 Yr	Study B	**–**	**✓**	**✓**	**✓**	**–**	**✓**	**✓**	**✓**	**✓**	**✓**
18–64 Yr	Study C	**–**	**–**	**✓**	**✓**	**–**	**✓**	**✓**	**✓**	**✓**	**✓**
18–64 Yr	Study B	**–**	**✓**	**✓**	**✓**	**–**	**✓**	**✓**	**✓**	**✓**	**✓**
≥60 Yr	Study C	**–**	**–**	**✓**	**✓**	**–**	**–**	**✓**	**✓**	**✓**	**✓**
≥65 Yr	Study C	**–**	**–**	**✓**	**✓**	**–**	**–**	**✓**	**✓**	**✓**	**✓**
	Study D	**–**	**✓**	**✓**	**✓**	**–**	**✓**	**✓**	**✓**	**✓**	**✓**

CBER, Center for Biologics Evaluation and Research (U.S. Food & Drug Administration). CHMP, Committee for Medicinal Products for Human Use (European Medicines Agency). Mo, months of age. y, years of age. SC, seroconversion licensure criterion (as defined in Materials and Methods section). GMR, geometric mean ratio licensure criterion (as defined in Materials and Methods section). Adult (18–60 years of age) CHMP licensure criteria were also applied to pediatric population and subjects 18–64 years old. Older adult (≥60 years of age) CHMP licensure criteria were also applied to subjects ≥ 65 years old.

**Table 5:** Percentages of subjects experiencing solicited local adverse events within seven days of any vaccination.

		6 Mo–5 y Full-Dose (n = 159)	6–17 y Full-Dose (n = 163)	18–64 y Full-Dose (n = 1636)	18–64 y Placebo (n = 387)	≥65 y Full-Dose (n = 1882)	≥65 y Placebo (n = 397)
Ecchymosis:	Any (%)	0	0	<1	0	<1	<1
	Grade I (%)	0	0	<1	0	<1	<1
	Grade II (%)	0	0	<1	0	0	0
Induration:	Any (%)	1	2	1	0	2	0
	Grade I (%)	1	2	<1	0	1	0
	Grade II (%)	0	0	<1	0	<1	0
	Grade III (%)	0	0	<1	0	0	0
Erythema:	Any (%)	3	1	<1	0	1	0
	Grade I (%)	3	1	<1	0	1	0
	Grade II (%)	0	0	<1	0	<1	0
	Grade III (%)	0	0	<1	0	0	0
Tenderness/Pain:	Any (%)	56	68	65	20	39	10
	Mild (%)	35	48	54	19	36	9
	Moderate (%)	20	19	11	<1	3	1
	Severe (%)	1	2	<1	<1	<1	0

Mo, months of age. y, years of age. Any, any measurable reaction at site of injection. Grade I, 25–50 mm; Grade II, 51–100 mm; Grade III, >100 mm. In subjects 6 months–5 years of age: mild tenderness/pain defined as, minor/light reaction to touch; moderate tenderness/pain defined as, cried/protested in response to touch; severe tenderness/pain defined as, cried when injected limb was moved. In subjects 6–17 years of age: mild pain defined as, does not interfere with daily activities; moderate pain defined as, interferes with daily activities; severe pain defined as, prevents daily activities. In subjects ≥ 18 years of age: mild pain defined as, does not interfere with daily activities; moderate pain defined as, interferes with daily activities or requires repeated use of non-narcotic pain medication; severe pain defined as, prevents daily activity or requires repeated use of narcotic pain medication. Placebo data derived from Study C. Data for subjects ≥ 18 years of age pooled from Studies B, C, and D.

**Table 6:** Percentages of subjects experiencing solicited systemic adverse events within seven days of any vaccination.

		6 Mo–5 y Full-Dose (n = 160)	6–17 y Full-Dose (n = 162)	18–64 y Full-Dose (n = 1636)	18–64 y Placebo (n = 387)	≥65 y Full-Dose (n = 1882)	≥65 y Placebo (n = 397)
Nausea:	Any (%)	–	13	11	11	7	6
	Severe (%)	–	1	<1	2	<1	<1
Fatigue:	Any (%)	–	27	25	21	19	19
	Severe (%)	–	1	1	2	<1	1
Myalgia:	Any (%)	–	30	17	11	11	8
	Severe (%)	–	0	<1	<1	<1	<1
Arthralgia:	Any (%)	–	13	12	9	10	9
	Severe (%)	–	0	<1	<1	<1	0
Headache:	Any (%)	–	22	25	23	15	16
	Severe (%)	–	0	1	2	<1	0
Malaise:	Any (%)	–	25	23	12	16	12
	Severe (%)	–	1	1	2	<1	<1
Loss of Appetite *:	Any (%)	18	14	9	9	6	6
	Severe (%)	0	1	<1	<1	<1	0
Fever:	≥38.0 °C (%)	16	4	1	2	1	<1
	≥40.0 °C (%)	1	0	<1	0	0	0

Mo, months of age. y, years of age. * Classified in subjects 6 months–5 years of age as, altered eating habits. In pediatric subjects ≤ 17 years of age, severity of systemic adverse events categorized according to Center for Biologics Evaluation and Research (CBER) grading scale: Grade 0 (none); Grade I (mild); Grade II (moderate); Grade III (severe). Any = Grades I–III inclusive. In adults and older adults ≥ 18 years of age, severity of systemic adverse events categorized according to CBER grading scale: Grade 0 (none); Grade I (mild); Grade II (moderate); Grade III (severe); Grade IV (potentially life-threatening). Any = Grades I–IV inclusive. Placebo data derived from Study C. Data for subjects ≥ 18 years of age pooled from Studies B, C, and D.

**Table 7:** Percentages of subjects experiencing unsolicited adverse events following any vaccination.

	6 Mo–17 y Full-Dose (n = 326)	18–64 y Full-Dose (n = 1683)	18–64 y Placebo (n = 398)	≥65 y Full-Dose (n = 1896)	≥65 y Placebo (n = 398)
Unsolicited AEs: Day 1–43 (%)	26	23	22	28	24
Vaccine-Related Unsolicited AEs: Day 1–43 (%)	4	7	6	9	7
Serious AEs ^†^ (%)	2	3	3	9	15
Vaccine-Related Serious AEs ^†^ (%)	0	<1	0	0	<1
AEs of Special Interest ^†^ (%)	0	<1	0	<1	2
New Onset of Chronic Disease ^†^ (%)	0	6	5	13	13
AEs Leading to Withdrawal ^†,^* (%)	<1	<1	<1	<1	<1
AEs Leading to Death ^†^ (%)	0	<1	0	<1	<1
AEs Requiring Medical Attention ^†^ (%)	34	38	37	55	55

Mo, months of age. y, years of age. AE, adverse event. ^†^ Events occurring throughout the entire duration of studies. * Data include deaths. Placebo data derived from Study C. Data for subjects ≥ 18 years of age pooled from Studies B, C, and D.

**Table 8:** Percentages of subjects experiencing any or at least possibly related unsolicited adverse events within 21 days of any vaccination (Day 1–43; MedDRA preferred terms occurring in ≥ 2% of any group).

MedDRA Preferred Term (%)	6 Mo–17 y Full-Dose Study A (n = 329)		18–64 y Full-Dose Study C (n = 1198)		18–64 y Placebo Study C (n = 398)		≥ 65 y Full-Dose Study C (n = 1197)		≥ 65 y Placebo Study C (n = 398)	
	All	V-R	All	V-R	All	V-R	All	V-R	All	V-R
Any AE	26	4	20	6	21	6	27	8	23	7
Arthralgia	1	<1	1	<1	2	<1	2	<1	<1	0
Diarrhea	2	0	<1	<1	1	0	1	<1	<1	<1
Fatigue	1	1	2	0	2	0	2	2	2	1
Headache	1	<1	2	<1	3	<1	2	<1	2	1
Injection Site Bruising	0	0	1	<1	2	2	2	2	2	2
Nasopharyngitis	4	0	0	0	0	0	0	0	0	0
Pyrexia	5	1	<1	<1	<1	<1	<1	<1	0	0
URT Infection	8	<1	<1	0	<1	0	1	<1	<1	0
Vomiting	2	1	<1	<1	0	0	0	0	0	0

MedDRA, Medical Dictionary for Regulatory Activities. Mo, months of age. y, years of age. AE, adverse event. URT, upper respiratory tract. All, all adverse events. V-R, adverse events at least possibly related to vaccination.

## Data Availability

The data presented in this report are available on request from the corresponding author.
